# Marijuana intoxication in a cat

**DOI:** 10.1186/s13028-018-0398-0

**Published:** 2018-07-11

**Authors:** Agnieszka Janeczek, Marcin Zawadzki, Pawel Szpot, Artur Niedzwiedz

**Affiliations:** 1 “Cztery Łapy i Ty” Veterinary Clinic, ul. Chabrowa 95 b, 52-200 Wysoka, Poland; 2 Department of Forensic Medicine, Wroclaw Medical University, ul. J. Mikulicza-Radeckiego 4, 50-345 Wrocław, Poland; 3 Department of Internal Diseases with Clinic for Horses, Dogs and Cats, Wroclaw University of Environmental and Life Sciences, pl. Grunwaldzki 47, 50-366 Wrocław, Poland

**Keywords:** Cat, Intoxication, Marijuana, THC

## Abstract

**Background:**

Cannabis from hemp (*Cannabis sativa* and *C. indica*) is one of the most common illegal drugs used by drug abusers. Indian cannabis contains around 70 alkaloids, and delta-9-tetrahydrocannabinol (delta-9-THC) is the most psychoactive substance. Animal intoxications occur rarely and are mostly accidental. According to the US Animal Poison Control Center, cannabis intoxication mostly affects dogs (96%). The most common cause of such intoxication is unintentional ingestion of a cannabis product, but it may also occur after the exposure to marijuana smoke.

**Case presentation:**

A 6-year-old Persian cat was brought to the veterinary clinic due to strong psychomotor agitation turning into aggression. During hospitalisation for 14 days, the cat behaved normally and had no further attacks of unwanted behaviour. It was returned to its home but shortly after it developed neurological signs again and was re-hospitalised. On presentation, the patient showed no neurological abnormalities except for symmetric mydriasis and scleral congestion. During the examination, the behaviour of the cat changed dramatically. It developed alternate states of agitation and apathy, each lasting several minutes. On interview it turned out that the cat had been exposed to marijuana smoke. Blood toxicology tests by gas chromatography tandem mass spectrometry revealed the presence of delta-9-tetrahydrocannabinol (THC) at 5.5 ng/mL, 11-hydroxy-delta-9-THC at 1.2 ng/mL, and 11-carboxy-delta-9-THC at 13.8 ng/mL. The cat was given an isotonic solution of NaCl 2.5 and 2.5% glucose at a dose of 40 mL/kg/day parenterally and was hospitalised. After complete recovery, the cat was returned to it’s owner and future isolation of the animal from marijuana smoke was advised.

**Conclusions:**

This is the first case of a delta-9-tetrahydrocannabinol intoxication in a cat with both description of the clinical findings and the blood concentration of delta-9-THC and its main metabolites.

## Background

Cannabis from hemp (*Cannabis sativa* and *C. indica*) is one of the most common illegal drugs used by drug abusers. Cannabis resin and oil are extracted from hemp plants. Indian cannabis contains approximately 70 alkaloids with delta-9-tetrahydrocannabinol (THC) being the most psychoactive compound. The concentration of delta-9-THC in the plant depends on the part of the plant, with the highest concentration in the feather crown and on the cultivation conditions. The concentration of delta-9-THC in the desiccated plant amounts to 12%, but when smoked not more than around 20% is transmitted into the smoke [1]. The cannabis resin contains approximately 10% of delta-9-THC, while cannabis oil (hashish oil) contains around 20% [2].

Animal intoxication occurs rarely and is most often accidental. Episodically, intentional exposure of an animal to marijuana may occur. According to the US Animal Poison Control Center (https://www.aspca.org/), cannabis intoxication mostly commonly affects dogs (96%) and more rarely cats (3%) but may also occur sporadically in other species. The most common cause of intoxication is an erroneous ingestion of a cannabis product, as well as secondary intoxication through exposure to marijuana smoke. The most popular form of cannabis, which is also eaten by pets is “marijuana butter” or so called “hash cookies”, acquired by placing parts of the plant in butter. As a result, the fat-soluble THC is extracted from the plant into the fat present in butter, reaching high concentrations [2].

This study presents a case of a cat intoxicated with marijuana. The study presents the clinical picture as well as the toxicology results of the patient’s blood that unequivocally confirmed its intoxication with delta-9-THC.

## Case presentation

A 6-year-old neutered female Persian cat presented to the “Cztery Łapy i Ty” veterinary clinic, Poland due to strong psychomotor agitation turning into aggression. The owner described the animal as “absent-minded”, disoriented, not reacting when called and behaving “strangely”. The cat walked around aimlessly, tried jumping into inaccessible areas, ran around the room, then hid and attacked people. This behavior was accompanied by loud meowing and hissing. The owner observed that prior to the aggression episodes, the cat had mydriatic pupils, which she believed showed “strong fear”. The cat demonstrated increased thirst, polyuria, attacks of gluttony mixed with periods of inappetence. During the attacks, the animal was not able to eat or drink by itself. According to the owner, the behavior of the animal made the members of the household afraid of it as it clapperclawed and bit its carers during the attacks. The periods of agitation and aggression were mixed with periods of normal behavior.

When the cat began to have sialorrhoea, sneezed and was not able to swallow, the owner sought veterinary assistance. The cat was examined clinically and then hospitalized for 3 weeks. A complete blood count and basic blood biochemical parameters were evaluated and found to be within normal range.

The cat was also clinically observed for symptoms of rabies over a 15-day hospitalisation period, but rabies was finally excluded. During the hospitalisation period, the cat behaved normally and had no attacks of unwanted behavior. The cat was then returned to its home.

After taking the cat home, the owner had to leave the pet with her partner for 3 weeks. When she returned, the symptoms of agitation/aggression and attacks had reoccurred but were more frequent and stronger. The day rhythm of the cat also changed—as it was mostly agitated at night.

Veterinary advice was sought again. Initially the cat was calm during the clinical examination, which included sampling of blood. The general condition of the cat was good. Respiration, pulse and rectal temperature were normal. The mucous membrane was pink and moist and the lymph nodes of normal size. The abdominal cavity was soft and painless on palpation and the patient showed no neurological abnormalities except for symmetrical mydriasis and scleral congestion. But during the consultation, the cat’s behavior changed diametrically. Alternate periods of agitation and apathy, each lasting several minutes, occurred. During the period of agitation, the cat jumped onto furniture, intensively explored the premises, walked into objects and stepped into a water bowl. In the quiet period, the cat sat in one place staring at one point and reacted poorly to external stimuli (mainly vocal; calling by the owner).

In the follow-up interview it turned out, that the aggressive behaviour commenced after longer absences of the owner of at least a couple of days, when the owner’s partner looked after the cat. The examining veterinarian asked the owner to contact her partner in order to identify possible causes of the medical condition of the cat. It turned out that the partner was marijuana-dependent and he admitted to have smoked marijuana while the cat’s owner was absent and to have exhaled marijuana smoke directly in the cat’s face “as a joke”.

The cat was given an isotonic solution of NaCl 2.5 and 2.5% glucose (Fresenius Kabi Polska Sp. z o.o., Warsaw, Poland) parenterally at a dose of 40 mL/kg/day for 2 days and was hospitalised in order to monitor the condition. During the 48-h hospitalisation, there were two periods of agitation and aggression. Sedatives were not applied as the attacks lasted less than a few minutes.

The blood sample underwent toxicological analysis. A screening enzyme-linked immunoassay analysis (ELISA) was used to screen for opiates, cannabinols, benzodiazepines, amphetamines and their derivatives as well as cocaine. A positive result was observed for cannabinols. Hence, further verification and quantitative tests were conducted with the use of gas chromatography tandem mass spectrometry (GC–MS) (Gas chromatograph: Aglient Technologies 6890N, Santa Clara, CA, USA; Mass spectrometer Aglient Technologies 5975B Santa Clara, CA, USA). The blood tests revealed the presence of delta-9-THC at 5.5 ng/mL, 11-hydroxy-delta-9-THC at a concentration of 1.2 ng/mL, and 11-carboxy-delta-9-THC at a concentration of 13.8 ng/mL.

Taking into account the clinical signs as well as the result of the toxicological tests, marijuana intoxication was diagnosed as the cause of the cat’s disease. The animal was returned to its owner and future isolation of the animal from marijuana smoke was advised.

## Discussion and conclusions

The toxicokinetics of delta-9-THC in cats has not been reported yet. In humans and laboratory animals, the metabolism of delta-9-THC depends mostly on the exposure route. Following inhalation, the concentration of THC rapidly increases in the blood, reaching its maximum concentration within a couple of minutes. When marijuana is ingested, the absorption of delta-9-THC is slower, and maximum blood concentration peak occurs 30–60 min after consumption. Delta-9-THC is rapidly metabolized by the liver, changed to 11-hydroxy-delta-9-THC and then oxidized to 11-carboxy-delta-9-THC. There are other delta-9-THC metabolites, but THC-OH and THC-COOH are the key cannabinoids used in the diagnosis of marijuana intoxication in humans [1].

Although studies have been conducted on neurological diseases [3, 4], inhibition of the cough reflex [5], or hypothermia [6] associated with delta-9-THC administration in cats, little is known about the feline metabolism of marijuana. We found that the metabolism of delta-9-THC in cats sees to resemble that in humans, as the same main metabolites were formed. In dogs, 8-hydroxsy-delta-9-THC is formed in addition to 11-hydroxy-delta-9-THC and 11-carboxy-delta-9-THC in the process of β-oxidation [7]. However, we did not assess this metabolite, and it remains unclear whether it is also formed in cats. The ELISA screening used to detect THCs in humans proved useful for cats as well.

The minimal toxic or lethal dose of delta-9-THC for a cat is unknown. In dogs, this dose must exceed 3 g/kg when administered orally [8] which is 1000 times higher than the dose causing behavioural disorders [2].

The clinical signs of an intoxication with delta-9-THC in cats differ from those in humans, and include disorders of consciousness, possibly leading to a coma, convulsions, ataxia, depression or agitation, anxiety, vocalization, hypersalivation, diarrhoea and vomiting, bradycardia or tachycardia, hypothermia and mydriasis [2].

The clinical signs of a marijuana intoxication in dogs have been reported by Janczyk et al. [9] but there are no similar studies in cats. Ninety-nine percent of canine patients show signs of a neurological disorder, while 30% have symptoms associated with the alimentary tract. Other studies show that the main symptoms of a delta-9-THC intoxication in dogs are mydriasis, urinary incontinence and hyperaesthesia [7]. In our study, the clinical findings were similar to those in dogs, although behavior disorders (aggression, disorientation), neurological disorders (mydriasis, disorders of movement coordination) and disorders of the alimentary tract (hypersalivation, gluttony attacks, and enhanced thirst) were the most prominent. Decrease in arterial blood pressure, bradycardia or lower respiratory rate, which have previously been reported [10] were not present in our case. This may be due to the exposure route as this affects the clinical presentation of the intoxication [11].

Cats may be prone to develop marijuana intoxication or cancers of the mouth and lymph nodes because of second-hand tobacco exposure [12]. When cats groom themselves, they lick up the toxins or carcinogens that accumulate on their fur. This grooming behaviour exposes the oral mucosa to the cancer-causing carcinogens, and it can intensify harmful effects of passive smoking [12]. Furthermore, in cases of deliberate exposure of pets to toxic substances, the owner, as well as the veterinarian must consider reporting the matter to relevant services.

The number of countries allowing legal use of marijuana for medical purposes has increased. In addition, some pet owners find marijuana to be a safe stimulant and smoke it for recreation at home in the presence of their pets. This case shows that the symptoms of delta-9-THC intoxication are unspecific and may resemble intoxication with other substances. It is advisable that the attending veterinarian consider toxicological tests and consider various causes of neurological dysfunction, outside standard causes, in order to diagnose and treat such cases.

This is the first laboratory confirmed published case of the clinical signs of a cat being intoxicated with delta-9-THC. Screening tests for use in humans proved useful for cats as well.
